# Case Report: Two Newly Diagnosed Patients With KBG Syndrome—Two Different Molecular Changes

**DOI:** 10.3389/fped.2021.649043

**Published:** 2021-09-17

**Authors:** Katarzyna Wojciechowska, Joanna Nurzyńska-Flak, Borys Styka, Magdalena Kacprzak, Monika Lejman

**Affiliations:** ^1^Laboratory of Genetic Diagnostic, Medical University of Lublin, Lublin, Poland; ^2^Department of Pediatric Hematology, Oncology and Transplantology, Medical University of Lublin, Lublin, Poland; ^3^Laboratory of Genetic Diagnostic, Children's University Hospital, Lublin, Poland; ^4^MedGen Medical Centre, Warsaw, Poland

**Keywords:** ankyrin repeat domain 11, neural development, KBG syndrome, intellectual disability, 16q24.2 microdeletion

## Abstract

Mutations or deletions of *ANKRD11* gene are responsible for the symptoms of KBG syndrome. The KBG syndrome is a rare genetic disorder which is inherited in an autosomal dominant manner. Affected patients usually have characteristic facial features, macrodontia of the upper central incisors, hand abnormalities, developmental delay and short stature. In the present article we would like to report a clinical and molecular case study of two patients affected by KBG syndrome. The diagnosis of the first patient was confirmed by the identification of the novel pathogenic variant in *ANKRD11* gene by next-generation sequencing. The second patient was diagnosed after the detection of a 16q24.2q24.3 deletion encompassing the *ANKRD11* gene microarray.

## Introduction

There are almost 200 patients with KBG syndrome reported in the literature since 1975 (1, 2). The prevalence of the disorder has not yet been established (3). The KBG syndrome was named after the initials of the last names of three original families reported by Herrmann et al. (4) in 1975.

Among the characteristic features of KBG syndrome are distinctive dysmorphic features, such as the triangular face, thin upper lip, prominent nasal bridge, synophrys, delayed bone age, short stature, and costovertebral anomalies. Patients may have intellectual disability, developmental delay; some of them may also suffer from seizures and EEG abnormalities. Symptoms are typically milder in females (3, 5). KBG syndrome is inherited in an autosomal dominant manner. Pathogenic variants in *ANKRD11* gene account for most cases (5, 6). Clinical diagnostic criteria include: (1) macrodontia of the upper central incisors, which is reported in more than 95% of cases and considered a distinctive trait of KBG syndrome; (2) characteristic facial features; (3) hand abnormalities, (fifth finger clinodactyly, clinical brachydactyly, or short tubular bones on the radiographic exam); (4) neurological problems, global developmental delay, and/or a seizure disorder, (5) bone age >2 SD below average; (6) costovertebral abnormalities, (abnormal curvature of the spine, cervical ribs, or vertebral/endplate defects); (7) postnatal short stature (as a height less than 3rd centile); and (8) occurrence of a first-degree relative affected by KBG syndrome (7). The patient should fulfill four or more of these eight criteria (7). The *ANKRD11* gene encodesankyrin repeat domain 11, a protein which contains multiple ankyrin domains^1^. Cell line studies indicate that Ankrd11 is a nuclear protein which regulates transcription by binding chromatin-modifying enzymes such as histone deacetylases. Castelo-Branco et al. (8) showed that modification of chromatin structure by histone acetylation is essential for the development and functioning of the nervous system and plays a particularly important role for neural precursors. In *Ankrd11*, knocked-down precursor cells of murine cortex decreased proliferation, reduced neurogenesis and aberrant neuronal positioning was observed (9). Furthermore, Gallagher et al. (9) show that mutated Ankrd11 in mice, perturbs neural development, and causes aberrant behavior. They also showed that Ankrd11 determines precursor proliferation, neurogenesis and neuronal positioning in the developing brain by regulating histone acetylation and gene expression. ANKRD11-deficient neurons displayed markedly reduced dendrite growth and branching as well as abnormal dendritic spine morphology (10). Barbaric et al. (11) associated *ANKRD11* with bone metabolism. They showed that homozygous Yoda mice are embryonic lethal, but that heterozygous mice with a missense mutation in the *Ankrd11* (*Ankrd11*^Yod/+^) survived into adulthood with altered bone metabolism and craniofacial abnormalities, reduced body size, as well as reduced bone mineral density. In many different genetic studies, scientists underline the role of *ANKRD11* gene which, when deleted, may induce intellectual disability, brain abnormalities and neurodevelopmental disorders including ASD (Autism spectrum disorder) (12, 13). In the present paper we would like to report a clinical and molecular case study of two patients affected by KBG syndrome. Both patients have uneventful family past histories and characteristic clinical phenotype. The diagnosis of the first patient was confirmed by the identification of the novel pathogenic variant in *ANKRD11* gene by next-generation sequencing. The second patient was diagnosed after the detection of a 16q24.2q24.3 deletion encompassing the *ANKRD11* gene by microarray.

## Clinical Features and Family History

### First Patient

**Figure 1:**
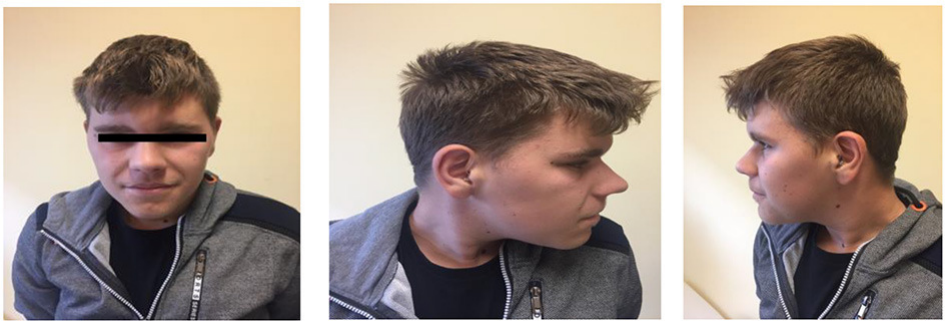
The face of the patient with a new pathogenic variant NM_001256182.1:c.5117delC NP_001243111.1:p.Pro1706LeufsTer13 in one copy of *ANKRD11* gene. Note a characteristic facial features: typical triangular face, wide eyebrows, synophrys, prominent nasal bridge, thick alae nasi.

**Figure 2:**
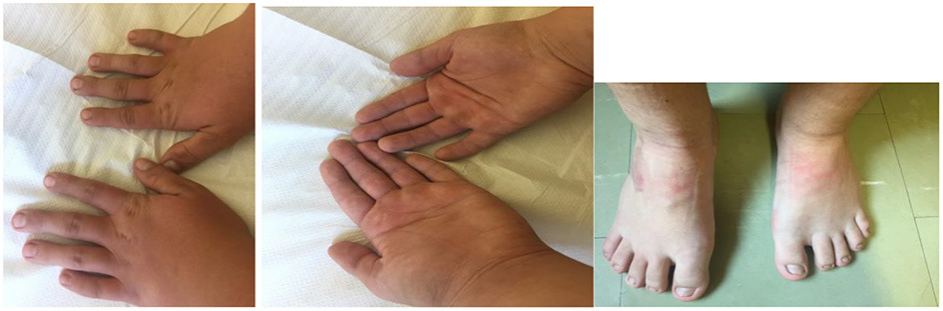
The hands and feet of the patient with a new pathogenic variant NM_001256182.1:c.5117delC NP_001243111.1:p.Pro1706LeufsTer13 in one copy of *ANKRD11* gene. Note a brachydactyly and wide fingers and toes.

The first patient was admitted to our Genetic Clinic at the age of nine. The boy was the first child of Caucasian, healthy, non-consanguineous parents. He had one healthy younger brother. Family history was insignificant and also the pregnancy was uneventful. The patient was born on time in a good condition with weight 4,060 g, length 57 cm and OFC 35 cm. He began to sit without support at the age of 6 months, he was standing at 11 months and walked well at 18 months. The boy did not vocalize till he was 3. At the age of 2, the patient had inguinal hernia surgery on the right side. The mother informed that because the boy has had speech problems and was under the care of logopedist. First evaluation by the psychologist at the age of 6 revealed that our patient had a moderate intellectual disability. All imaging tests, such as computed tomography of the head, abdominal ultrasound, echocardiography, Doppler ultrasound of carotid arteries, did not show abnormalities. We ordered microarray testing which later did not show any clinically significant change. During the consultation at the age of 13 years, the boy was 156 cm tall (50th percentile), and his weight was 77.5 kg. He was diagnosed with mild intellectual disability, hyperinsulinemia and mild bilateral sensorineural hearing loss (our patient was wearing hearing aids in both ears). A routine echocardiographic examination showed PFO, VSD and PDA. A testicular ultrasound showed bilateral cryptorchidism and hydrocele in the right testicle. In our Genetic Clinic, we took the blood from the patient and ordered Whole Exome Sequencing. New pathogenic variant NM_001256182.1:c.5117delC NP_001243111.1:p.Pro1706LeufsTer13 was found in one copy of *ANKRD11* gene. Both parents were tested and none of them was a carrier. At the age of 14, our patient was 161 cm tall, his OFC was 56 cm and he weigh 79 kg. He was diagnosed with moderate intellectual disability, he did not speak full sentences. He still had hyperinsulinemia, which was treated with metformin. His bilateral sensorineural hearing loss did not progress. According to his mother, he was very polite, he liked biking and playing football with friends. He had significant dysmorphic facial features which are presented on the pictures in the Figures 1, 2.

The patient will stay under the care of endocrinologist, audiologist and psychologist. He has not undergone growth hormone treatment. He will also come to our Genetic Clinic for the annual evaluations. We plan to do blood tests each year and abdominal ultrasound as a form of cancer surveillance.

### Second Patient

**Figure 3:**
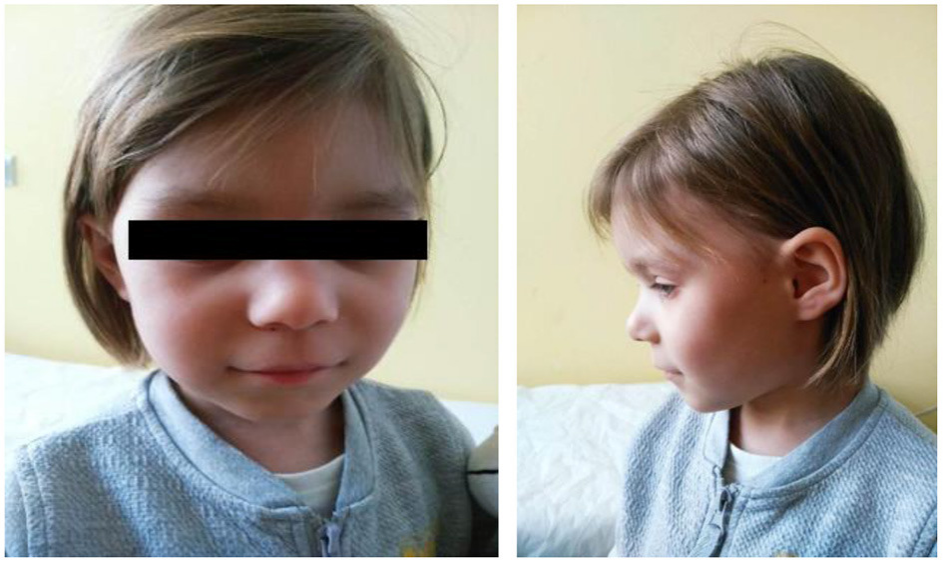
The face of the patient with a 16q24.2q24.3 microdeletion. Note a characteristic facial features: wide eyebrows, bulbous nose with prominent nasal bridge, thin upper lip, long philtrum.

**Figure 4:**
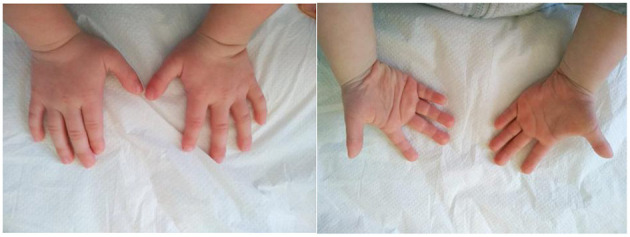
The hands of the patient with a 16q24.2q24.3 microdeletion. Note a brachydactyly.

The second patient was admitted to our Genetic Clinic at the age of 4. She was the first daughter of a Caucasian, non-consanguineous couple. She had a healthy 2-year-younger brother. Family history was insignificant; however, her mother was diagnosed with Hashimoto disease. The patient was born on time in a good condition with low birthweight 2,345 g, length 47 cm and OFC 32 cm. The abdominal ultrasound and echocardiography did not show any abnormalities. She began sitting without support at the age of 14 months and walked well at 21 months. She started to speak at the age of 32 months. She was advised to visit a Genetic Clinic at the age of 4 because of short stature and developmental delay. At the time of the first visit, her growth parameters were below the age norm (90 cm), she had a hoarse voice, mild dysmorphic facial features, synophrys, highly arched palate, crowded teeth, valgus feet, laxity of joints. She was diagnosed with asthma and subclinical hypothyroidism. She had also a tendency for constipation. After the visit, we ordered microarray testing which later showed a deletion in the region 16q24.2q24.3 (it contained 1,543 Mbp). Deletion contains 37 genes i.a.: *BANP* (611564), *ZNF469* (612078), *ZFPM1* (601950), *IL17C* (604628), *CYBA* (608508), *MVD* (603236), *SNAI3* (612741), *RNF166* (617178), *CTU2* (617057), *PIEZO1* (611184), *CDT1* (605525), *APRT* (102600), *GALNS* (612222), *TRAPPC2L* (610970), *CBFA2T3* (603870), *ACSF3* (614245), *CDH15* (114019), *ANKRD11* (611192) and it contains the region of the known 16q24.3 microdeletion syndrome (KBG syndrome). Subsequently, we tested the parents who were not carriers of that deletion. At the age of 5, our patient was 100.5 cm tall; her OFC was 49 cm and her weight was 14 kg. She attended kindergarten, she had phoniatric voice rehabilitation and started to speak more using full sentences. She was monitored by gastroenterologist, audiologist and endocrinologist. Our patient at the age of 5 years is presented on the pictures in the Figures 3, 4.

Short stature is one of the symptoms of KBG syndrome. The results of preliminary tests suggest that growth hormone treatment may increase the height potential of the patients, therefore she may need growth hormone therapy in the future. She will be evaluated annually by endocrinologist, audiologist and clinical geneticist. She will have blood tests and abdominal ultrasound every year.

In Table 1, we compare the characteristic features of KBG syndrome, which are listed in OMIM, and features of our patients.

**Table 1:** Characteristic features of KBG syndrome, which are listed in OMIM, and features of our patients.

**Characteristic features of** ** KBG syndrome (OMIM)**	**Patient no** ** 1 male**	**Patient no** ** 2 female**
Short stature	+	–
Microcephaly	–	–
Round face early in life	+	–
A triangular face later in life	–	+
Long philtrum	+	+
Large prominent ears	–	–
Hypertelorism	–	–
Telecanthus	–	–
Long palpebral fissures	+	–
Broad bushy eyebrows	+	+
Anteverted nares	–	–
Hypoplastic alae nasi	–	–
Macrodontia	–	–
Wide upper central incisors	–	–
Ridged teeth	–	–
Fused incisors	–	–
Oligodontia	–	–
Cervical rib fusion	–	–
Accessory cervical ribs	–	–
Cryptorchidism	–	+
Delayed bone maturation	–	–
Vertebral body fusion	–	–
Vertebral arch abnormalities	–	–
Thoracic kyphosis	–	–
Clinodactyly	–	–
Decreased hand length	–	+
Syndactyly	–	–
Simian crease	–	–
Broad bushy eyebrows	+	+
Low anterior hairline	–	–
Low posterior hairline	–	+
Developmental delay	+	+
Mental retardation	–	+
EEG anomalies	–	–
Seizures	–	–

## Methodology

### Genetic Findings

**Figure 5:**
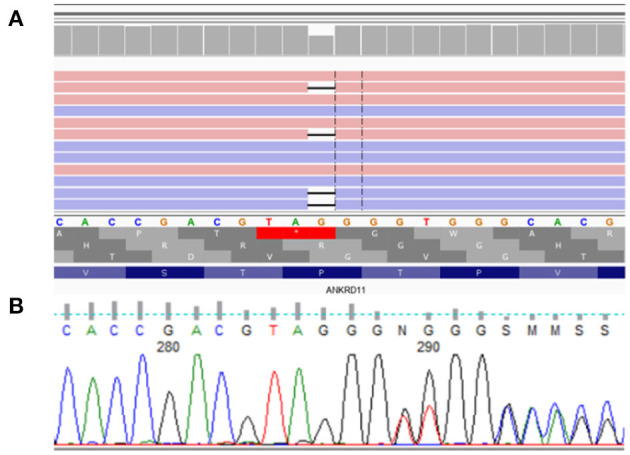
*De novo* heterozygous frameshift variant NM_001256182.1:c.5117delC NP_001243111.1:p.Pro1706LeufsTer13 in exon 10 (of 14) of *ANKRD11*. **(A)** presents analysis from NovaSeq sequencing platform, **(B)** confirmation of detected variant by Sanger sequencing.

Peripheral blood samples were collected from the patients and their parents. DNA from whole blood sample was isolated using Prepito TM. The whole exome sequencing library was prepared using SureSelect XT Library preparation Kit (from Agilent). The sample was sequenced with Illumina technology on NovaSeq6000 sequencer with 2 × 100 bp reads. The obtained QC value was 92.89% for Q30. Demultiplexing of the sequencing reads was performed with Illumina bcl2fast (2.19). Adapter was trimmed with Skewer version 0.2.9 (14). The reads were aligned to GRCh37/hg19 reference sequence using BWA-MEM. Read duplicates were removed using Picard 2.18.2 (http://broadinstitute.github.io/picard/). The results are presented in the Figure 5. Variant call was performed with GATK v4.0.3.0 HaplotypeCaller (15) and FreeBayes (v1.2.0-2-g29c4002). Variants have been annotated with databases: (i) VEP97 (16): annotations Sift, Polyphen2, (ii) dbNSFPv4.0 (17) annotations: MutationAssessor, MutationTaster, DANN, FATHMM, (iii) ESP6500, (iv) GnomAD, (v) dbSNP, (vi) ClinVar and (vii) 1,000 Genomes. CNV analysis was performed using XHM (18). The presence of variants detected by next generation sequencing was confirmed by Sanger sequencing.

Molecular analysis of the *ANKRD11* gene showed *de novo* heterozygous frameshift variant NM_001256182.1:c.5117delC NP_001243111.1:p.Pro1706LeufsTer13 in exon 10 (of 14) of *ANKRD11*.

According to ACMG Standards and Guidelines (19)^2^, variant NM_001256182.1:c.5117delC NP_001243111.1:p.Pro1706LeufsTer13 in exon 10 of *ANKRD11* is classified as strongly pathogenic because it is a null variant (frame-shift) which is a known mechanism of disease, associated with KBG syndrome (PVS1, PM1). It was not found in GenomAD exomes and genomes (PM2), it has computational verdict based on 1 pathogenic prediction from phyloP vs. no benign predictions (PP3), and the variant is *de novo* mutation (both maternity and paternity confirmed) in a patient with the disease and has no family history (PS2).

**Figure 6:**
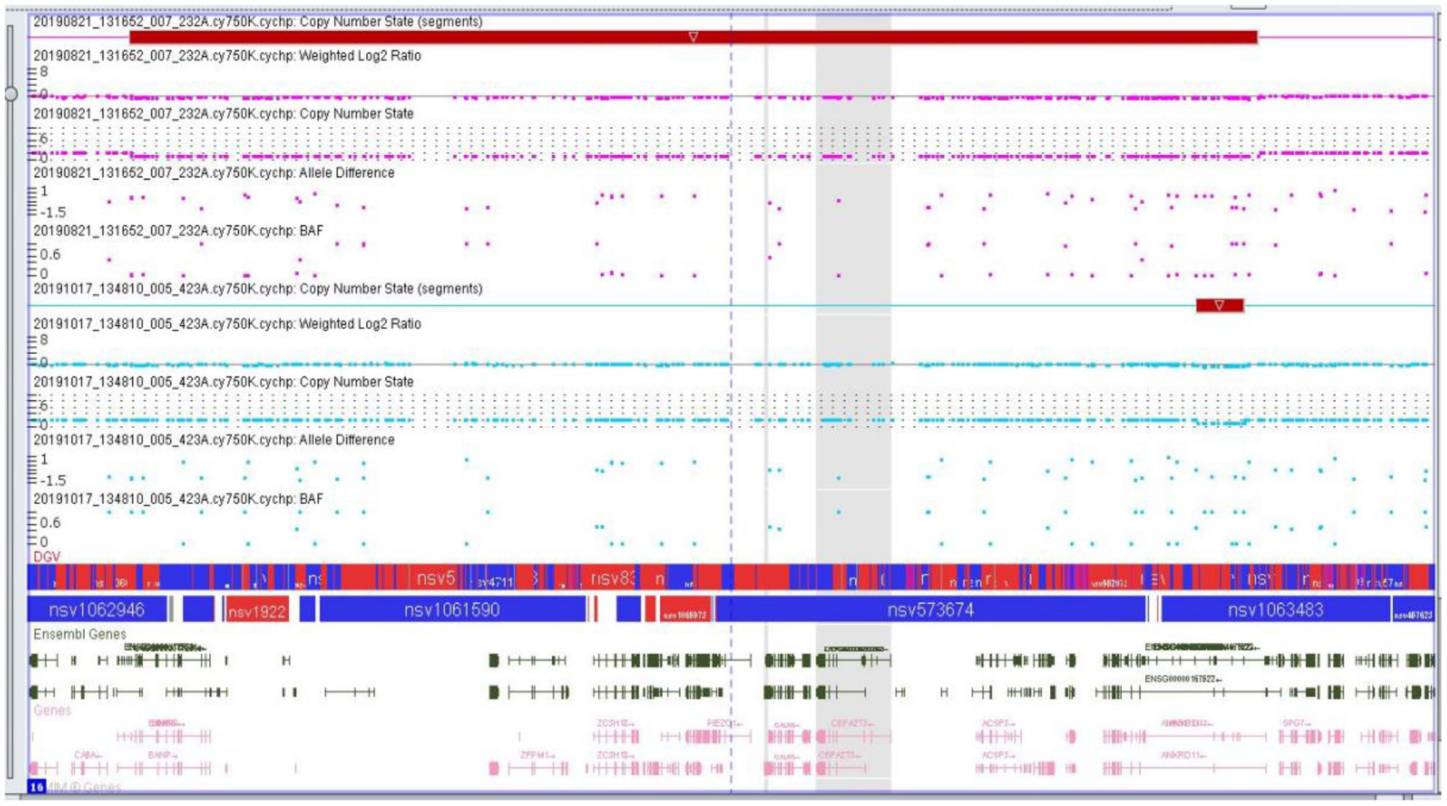
Microarray result of the second proband showing genomic imbalance- deletion in the 16q24.2q24.3 chromosome region.

The microarray analyses were performed by using the CytoScan 750K array (750,000 oligonucleotide probes and 200,000 probes) identifying the polymorphism of a one single nucleotide polymorphism (SNP) (Applied Biosystems, Thermo Fisher Scientific, Waltham, MA, USA). A total of 250 ng of the genomic DNA was evaluated in accordance with the manufacturer's protocols. The study was based on an analysis of scanned data files that were generated with the Chromosome Analysis Suite v 3.3 (ChAS, Thermo Fisher Scientific, Waltham, MA, USA). Furthermore, the copy number of altered regions (CNAs) was calculated, and the data were normalized to the reference model (Thermo Fisher Scientific, Waltham, MA, USA) of baseline reference intensities NA 33 (hg19/CRCh37). The copy number states (CNS) and their breakpoints were determined with the use of the Hidden Markov Model (HMM) software package. The threshold level of log2 ratio ≥0.5 and ≤ 0.5 were used, respectively, for the categorization of the altered chromosomal regions as copy number variation (CNV) gains and losses. The minimal number of probes was applied to determine the CNAs: 50 probes for duplication (gain) and 25 probes for deletion (loss). To further identify the genes involved in the CNVs, two databases were applied: the UCSC database (http://genome.ucsc.edu) and Ensemble (http://www.ensembl.org). The results are presented in the Figure 6.

## Discussion

KBG syndrome is caused by haploinsufficiency of the *Ankyrin repeat domain-containing protein 11* (*ANKRD11*) gene resulting from either loss-of-function pathogenic variants in the *ANKRD11* gene or microdeletions in chromosome 16q24.3, which includes *ANKRD11* (1, 12, 20, 21). ANKRD11 is a chromatin regulator and it displays a dual function in transcriptional activation and suppression. It contains two transcription repression domains and one activation domain. ANKRD11 by interacting with histone deacetylases (HDACs) and histone molecules participates in the process of transcription inhibition, resulting in the inhibition of ligand-dependent transcription. In a different cellular context, ANKRD11 may activate gene transcription by interaction with histone acetyltransferases (HATs) (22). Ka and Kim (10) showed that ANKRD11 regulates pyramidal neuron migration and dendritic differentiation in the developing mice's cerebral cortex. Using an *in utero* manipulation approach, they found that *Ankrd11* knockdown delayed radial migration of cortical neurons. ANKRD11-deficient neurons had significantly reduced branching and dendrite growth as well as abnormal dendritic spine morphology. In 2010, Willemsen et al. (13) presented four patients with distinctive facial features, structural anomalies of the brain, variable cognitive impairment, autism spectrum disorder and seizures: all of them had *de novo* microdeletions of 16q24.3. They proposed that these patients represent a novel and distinctive microdeletion syndrome, which may be characterized by facial dysmorphisms, brain abnormalities, autism spectrum disorder and a variable cognitive impairment. They also suggested that haploinsufficiency of ANKRD11 and/or ZNF778 may contribute to this phenotype. Two years later, Isrie et al. (12) presented two patients with short stature, cognitive impairment and dysmorphic features carrying a small deletion in the 16q24.3 region containing the ANKRD11 gene. These observations confirmed the findings of Willemsen et al. (13) and further strengthen the hypothesis that ANKRD11 is a candidate gene for autosomal dominant (syndromic) intellectual disability. Two patients presented by Isrie et al. (12) had distinctive facial features, such as a relatively small nasal bridge with mild synophrys and a bulbous nose tip. The stature of both patients was relatively short. They did not have structural brain malformations or heterotopias, although epilepsy at childhood age was present in the second patient. Both of our patients presented similar phenotype. Interestingly, the patient carrying the largest deletion, described by Willemsen et al. (13), had a hearing impairment. Hearing loss was also diagnosed in our first patient, who needed a hearing aid, and a slight unilateral impairment was diagnosed in our second patient. We did not observe structural brain malformations or heterotopias in our first patient. The MRI of the second patient's brain has not yet been performed. The comparison of clinical features of patients with ANKRD11 loss of function variants with those with 16q24.3 microdeletions made by Ockeloen et al. (23) reveals that the frequency of congenital anomalies, seizures and behavioral problems appeared to be similar in both groups. Chen et al. (24) presented a patient with heterozygous c.1366_1367dup (p.K457Rfs^*^54) variant in exon 9 of *ANKRD11* who had a typical symptoms of KBG syndrome: characteristic dysmorphic features, dental crowding, brachydactyly, left-skewed caudal vertebrae. Novara et al. (25) presented 12 cases with a 16q24.2q24.3 deletion (*de novo* in 11 cases), ranging from 343 kb to 2.3 Mb. In 11 cases, the deletion involved the *ANKRD11* gene and in one case only flanking genes upstream to it was deleted. The results show that the patients who had more severe neurological phenotype and congenital heart defects, besides *ANKRD11*, had also a deletion of *ZFPM1, CDH15* and *ZNF778* gene. The researchers suggest also that the presence of thrombocytopenia and astigmatism should be considered in distinguishing between 16q24 microdeletion syndrome and KBG syndrome. *ZFPM1* haploinsufficiency causes severe disturbances during developmental stages, especially affecting the eyes, the heart and the hematopoietic system (26). However, although the gene encodes a nine-zinc-finger transcriptional regulator required for proper differentiation and maturation of both megakaryocytes and erythroid precursors, patients presented by Novara et al. (25) did not show bleeding disorders. Interestingly, *ANKRD11* itself is also expressed in bone marrow at a high level, but none of the 38 presented patients showed thrombocytopenia. Likewise, none of our patients had bleeding disorders. *ZFPM1* is also crucial for correct eye development through the interaction with GATA factors, and its haploinsufficiency seems to be associated with severe astigmatism, what was presented in several patients. Our second patient with a deletion on in the region 16q24.2q24.3 did not have any vision problems.

Novara et al. (25) reported a higher frequency of congenital heart defects (CHDs) in the group of patients with 16q24.3 microdeletion (about 40% of cases) in comparison with KBG subjects (10%). Haploinsufficiency for *ZNF778*, which is extensively expressed in the brain and the heart, may contribute to the higher frequency of ventricular septal defects present in the patients with 16q24 microdeletion syndrome (25). However, not every patient with 16q24 microdeletion containing *ZNF778* has a congenital heart defect. Our second patient was in a group of children without any structural heart abnormalities. Among the deleted genes, she had a deletion of *CDH15* which encodes a calcium-dependent cell adhesion molecule, cadherin 15. The observation of a worse neurological phenotype for larger deletions suggests a possible role for *CDH15* in regulating proper central nervous system embryogenesis. In the future, she will need psychological evaluation and her intelligence quotient should be measured.

The dental dysmorphisms reported in patients with 16q24.3 microdeletion by Novara et al. (25) are very similar to those described for ANKRD11 haploinsufficiency, albeit occurring with a lower frequency. Our patient with deleted region 16q24.2q24.3 did not have any dental abnormalities.

Based on the observation of 39 patients with KBG syndrome of Goldenberg et al. (27), the authors reported that macrodontia should no longer be considered an obligatory feature of the syndrome. We did not observe it in our second patient. In a group where 19 patients had a deletion of a 16q24.3 region and 20 patients had pathogenic variants in *ANKRD11*, the studies showed that patients may have autonomous life in adulthood and autism is less frequent than previously reported; it is convergent with the observations we made on our patients. The authors described two new clinical findings such as precocious puberty and a case of malignancy (27). Those findings may have a potential impact on the follow-up of our patients. Neilsen et al. (28) found that ANKRD11 can be a novel p53-interacting protein that enhanced the transcriptional activity of p53. ANKRD11 expression was shown to be downregulated in breast cancer cell lines. Moreover, Behnert et al. (29) reported a 17-year-old patient who was diagnosed with a left-sided paratesticular extrarenal malignant rhabdoid tumor. Genetic testing identified a constitutional *de novo* 16q24.3 microdeletion leading to the loss of the entire *ANKRD11* locus. It has been speculated that haploinsufficiency of *ANKRD11* may lead to increased cancer risk in patients with KBG syndrome. The results show that *ANKRD11* may be a tumor suppressor gene. At present, no specific measures for cancer surveillance can be recommended for KBG syndrome patients, however, more data are needed to confirm this.

## Conclusion

In summary, KBG syndrome remains still under-diagnosed (27). Research suggests that the pathogenic variants of *ANKRD11* gene are a common Mendelian cause of developmental delay and that it is one of the major genes associated with intellectual disability. Our data confirm that the phenotypes of patients with KBG syndrome differ between each patient. Further additional patient reports will certainly advance the delineation of a specific phenotype caused by haploinsufficiency of ANKRD11. In the future, more data are also needed to confirm or deny an increased cancer risk in patients with KBG syndrome.

## Data Availability Statement

The datasets presented in this study can be found in online repositories. The names of the repository/repositories and accession number(s) can be found below: NCBI ClinVar SCV001571236.

## Ethics Statement

Written informed consent was obtained from the minor(s)' legal guardian/next of kin for the publication of any potentially identifiable images or data included in this article.

## Author Contributions

KW drafted the manuscript. KW and JN-F contributed to the clinical data acquisition. BS, MK, and ML contributed to the analysis and genetic evaluation. ML critically revised the manuscript. All authors contributed to the article and approved the submitted version.

## Conflict of Interest

The authors declare that the research was conducted in the absence of any commercial or financial relationships that could be construed as a potential conflict of interest.

## Publisher's Note

All claims expressed in this article are solely those of the authors and do not necessarily represent those of their affiliated organizations, or those of the publisher, the editors and the reviewers. Any product that may be evaluated in this article, or claim that may be made by its manufacturer, is not guaranteed or endorsed by the publisher.

## Footnotes

^1^https://ghr.nlm.nih.gov/gene/ANKRD11.

^2^https://www.acmg.net/ACMG/Medical-Genetics-PracticeResources/Technical_Standards_and_Guidelines.aspx.

## Abbreviations

ACMG: The American College of Medical Genetics and GenomicsCDH15 Cadherin 15
MRI: Magnetic Resonance Imaging
OFC: Occipital Frontal Circumference
PDA: Patent Ductus Arteriosus
PFO: Patent Foramen Ovale
PVS1: Pathogenic Variant Sequence 1
VSD: Ventricular Septal Defect
ZFPM1: Zinc Finger Protein Multitype 1
ZNF778: Zinc Finger Protein 778.
